# What's really wrong with cognitive behavioral therapy for psychosis?

**DOI:** 10.3389/fpsyg.2015.00323

**Published:** 2015-03-27

**Authors:** Neil Thomas

**Affiliations:** ^1^School of Health Sciences, Swinburne UniversityMelbourne, VIC, Australia; ^2^Monash Alfred Psychiatry Research Centre, Monash UniversityMelbourne, VIC, Australia

**Keywords:** psychosis, cognitive-behavioral therapy, schizophrenia, hallucinations, delusions, efficacy, evidence-based practice, dissemination

## The CBT debate

For persons with medication-refractory psychotic experiences such as auditory hallucinations, a psychological therapy referred to as cognitive behavioral therapy for psychosis (CBTp) has emerged as the standard recommended treatment in clinical practice guidelines (e.g., Kreyenbuhl et al., 2010; National Institute for Health and Clinical Excellence [NICE], 2014). However, the past few years have seen impassioned debate regarding the endorsement of CBTp as evidence-based practice, with some arguing that evidence in its favor has been “oversold” (McKenna and Kingdon, 2014). As a follow up to their earlier controversial review (Lynch et al., 2010), which claimed no evidence that CBTp was effective in “well-conducted” trials, a recent meta-analysis by Jauhar et al. (2014) drew the only slightly less pessimistic conclusion that CBTp's therapeutic effect was only in the small range. Coinciding with the continuing recommendation of CBTp for routine provision in the 2014 NICE guidelines, this has led to debates published in several journals, and a flurry of further meta-analyses analysing different permutations of trial characteristics and measures. These meta-analyses have formed the more optimistic conclusions that CBTp shows good effects for hallucinations (van der Gaag et al., 2014), for overall psychotic symptoms in people with persisting symptoms (Burns et al., 2014), and in direct contrasts with other interventions (Turner et al., 2014).

As a battle fought with meta-analysis, debate has focused on which data should be included in effect size calculations: for example, whether trials with different intervention targets should be included, conducted during acute psychosis should be mixed with persisting psychosis, and whether both group and one-to-one format intervention should be included (Birchwood et al., 2014; Burns et al., 2014; Mueser and Glynn, 2014; Peters, 2014). There has also been criticism of an excessive focus on overall psychotic symptom severity at post-treatment as the primary outcome (e.g., on measures like the Positive and Negative Symptom Scales, PANSS), when there is stronger evidence for effects on specific symptom measures (Peters, 2014; van der Gaag et al., 2014), and at follow-up time points (Peters, 2014), and when, in any case, CBTp primarily targets the emotional impact of psychotic experiences rather than their presence or frequency (Birchwood et al., 2014).

## Beyond the effect size debate

This debate has been valuable in drawing attention to the need for critical thinking in this field. However, the present paper argues that debate about which data to include in analyses, and how to interpret them, needs to consider some fundamental limitations of using broad CBTp protocols as a focus of study, and highlights that this whole debate has overlooked some potentially more fundamental limitations of what CBTp provides as an intervention for routine practice.

The term “cognitive behavioral therapy for psychosis” emerged in the 1990s within the context of a number of researchers beginning to study the application of psychological methods to help people manage their psychotic symptoms. At this time, this was helpful in grouping together a number of similar approaches, united by a pragmatic focus on reducing the impact of symptoms, and use of methods from a cognitive behavioral tradition, such as training in different skills in responding to symptoms, and restructuring distress-related appraisals. Hence, the operationalization of a CBTp served a useful purpose in defining an emerging literature, in developing therapy manuals for RCTs, and, ultimately, in providing data for the meta-analyses which have informed guidelines. However, as the field has progressed, the concept of CBTp has become less relevant and more limited, both as an object of scientific enquiry, and as a model of provision in practice.

## Limitations of the CBTp protocol as an object of scientific enquiry

One of the key limitations of using CBTp as a trial protocol is the level of within-trial heterogeneity of therapy delivery. Trials have usually included persons with some combination of positive symptoms (hallucinations, delusions, or both), often in combination with negative symptoms and emotional symptoms, which means a variety of therapy targets are possible. Intervention protocols also describe a variety of therapeutic methods (e.g., coping enhancement, modification of delusions, relapse prevention), employed on the basis of an individualized case formulation, which makes it difficult to derive conclusions about the helpfulness and importance of component methods. In contemporary practice, as further intervention methods are incorporated, such as the use of mindfulness and imagery, CBTp represents an increasingly varied smorgasbord of intervention methods used in various combinations according to the person's presentation.

One consequence of this within-trial variability is that CBTp trials have required outcome measures sufficiently broad to capture the expected (variable) outcome domains. Whilst viable for broad outcomes such as quality of life and service use, within-trial variability reduces sensitivity to more direct but individualized outcomes such as hallucination- or delusion-related distress, compliance with command hallucinations, and interference of specific symptoms with social functioning. This has resulted in an overreliance on omnibus psychosis measures like the PANSS, which are so broad and indirect that they may measure more things that are not targeted by the intervention than things that are (Thomas, 2014). Such measures are inevitably going to lack sensitivity to individual outcomes, and, subsequently, provide trials with little more than a fairly insensitive test of a null hypothesis that CBTp does nothing at all. It would be misleading to consider the magnitude of effect sizes produced from these data as especially meaningful.

A second consequence of using an individualized formulation-based approach is that trials do not reveal which of the many intervention components that were included in the intervention contributed to outcome. For example, a recent review of psychological intervention methods for auditory hallucinations found that relatively little could be concluded about the relative usefulness of specific components of CBTp used with voices, with many assumptions untested, and a need for much more study at this level (Thomas et al., 2014).

A related, third, limitation regards the amenability of CBTp as a protocol for RCT designs contrasting with control conditions that match for therapist time, analogous to the use of placebo control in drug trials, argued by Lynch et al. to be a feature of “well-conducted” trials. In principle, this trial design could provide a strong experimental approach to examining therapeutic technologies: it would work well if there were a more tightly operationalised intervention, and more tightly defined outcome measure, to test whether the specific content of the intervention contributes over more contextual factors in delivery. However, this degree of specificity is not provided by the broad CBTp protocol, where the intervention arm involves a variably utilized package of intervention methods rather than a specific intervention. This, combined with broad outcome measurement, and the reality that psychological therapy control conditions have more active components than placebo/expectancy alone, means that such designs are likely to be very difficult to power adequately.

## Limitations of CBTp as a model of best-practice therapy

Meanwhile, the second key issue regards how well the broad CBTp model, which has been the focus of trials, informs practice. Ultimately, the purpose of developing any intervention is to see it delivered. In the 1990s, CBTp was exciting in providing promise to target symptoms which were being left to pharmacotherapy alone. However, whilst clinical practice guidelines have been encouraging routine provision of CBTp since 2002, there continues to be discussion of it failing to be delivered (e.g., Farhall and Thomas, 2013; Nordentoft and Austin, 2014; van der Gaag, 2014). In explaining this, it is often argued that services need to better prioritize psychological interventions. However, what does CBTp actually offer? CBTp mainly provides a framework for adapting existing cognitive and behavioral methods to psychosis, thereby primarily being suited to delivery by practitioners with advanced levels of cognitive-behavioral skill, typically clinical psychologists. Indeed, the competency framework described for CBTp (Roth and Pilling, 2013) indicates a high and exclusive bar for delivery. In practice, the CBTp intervention we have validated as evidence-based practice within RCTs outlines a treatment protocol requiring such high prerequisite skill that it can only be used by a small—and expensive—segment of the mental health workforce.

Furthermore, without understanding which components of the CBTp black box are useful, this creates a challenge of either disseminating a simplified version of CBTp to less thoroughly trained practitioners, or increasing the workforce of skilled therapists. Both have been attempted with less success than would be hoped. Early initiatives such as THORN in the UK have had relatively poor success in leading to changes in delivery (Couldwell and Stickley, 2007), and it is notable that the most recent descriptions of “low intensity” CBT for psychosis have not, in fact, utilized elements of CBTp, but, rather, behavioral activation and graded exposure (Waller et al., 2013). Meanwhile, recent UK pilots have examined whether it is possible to increase delivery of CBTp by boosting the workforce of CBT practitioners. Jolley et al. (2015) recently described the outcomes in a demonstration site in which a total of 10 additional whole time equivalent NHS staff focused on delivering CBTp to psychosis services with approximately 7000 service users. They reported being successful in leading to a threefold increase in persons delivered CBTp with evidence for good outcomes and high satisfaction in participants within a year. However, with delivery reported to increase at a rate of approximately 20 cases per whole time equivalent therapist per year, the total increase in delivery would have represented less than 3% of the overall caseload. This suggests that, even with significant investment and prioritization, only quite low absolute levels of delivery can be realized, illustrating challenges in meeting consumers' treatment needs in a widespread way using CBTp as a model.

## What can we learn from this for the ongoing development of interventions?

These limitations highlight issues, not of CBTp lacking efficacy, but of it providing a research focus not particularly revealing of its component methods or specific effects, and producing an all-or-nothing therapeutic technology that is not well-suited to being widely accessed. It is important to recognize when the CBTp protocol has uses and when it is limited. In research, it works well as a protocol representing current best-practice expert psychological therapy, something useful, for example, in contrasts with routine care to address cost-effectiveness questions, or when considering applications in particular contexts, such as with persons not taking medication (e.g., Morrison et al., 2014). However, its operationalization is not tight enough to know what exactly is working. Likewise, in practice, CBTp provides a useful operationalization of how to work psychologically with psychosis for persons already fluent in CBT, but is less accessible as an intervention for practitioners new to CBT to learn.

So what can we learn from this in advancing the field of psychological interventions for psychotic symptoms such as auditory hallucinations? Being much broader than, for example, cognitive therapy for depression, CBTp is not well-suited to dismantling trials. However, there is value in individual examination of more discrete intervention components, both old and new, that can be isolated more precisely, which has been showing some success with delusions (see Freeman, 2011; Thomas et al., 2014). This lesson is important not just for cognitive behavioral interventions, but also for the direction of ongoing research into new broad therapy paradigms being applied to psychotic experiences, such as acceptance and commitment therapy (e.g., Thomas et al., 2013) which may run into similar challenges in future.

In doing this, it appears particularly important to be focusing more attention to simple methods amenable to being widely delivered in practice. In considering the challenges that have been faced in disseminating CBTp, there is potential value in questioning the approach of starting with what expert therapists can do, and seeking to disseminate it, and instead starting with what patients need (e.g., Byrne and Morrison, 2014), and what mental health practitioners and services can deliver, and building interventions around this. This might include greater focus on consumer-defined ideas of personal recovery, and consumer-identified subjective and functional impacts of psychotic experiences found most problematic; as well as considering what can be provided by current mental health practitioners, and new elements such as a growing peer workforce (e.g., Dent-Pearce et al., 2015) and digital technologies (Alvarez-Jiminez et al., 2014).

### Conflict of interest statement

The author declares that the research was conducted in the absence of any commercial or financial relationships that could be construed as a potential conflict of interest.
